# DA-REPOCH Versus R-CHOP for the Treatment of Activated B-Cell Subtype Diffuse Large B-Cell Lymphoma: A Community Center Experience

**DOI:** 10.7759/cureus.11714

**Published:** 2020-11-26

**Authors:** Phillip Knouse, Edward Nabrinsky, Ronald L Sirota, David Hakimian, Jacob Bitran

**Affiliations:** 1 Hematology and Medical Oncology, Advocate Lutheran General Hospital, Park Ridge, USA; 2 Internal Medicine, Advocate Lutheran General Hospital, Park Ridge, USA; 3 Pathology, Advocate Lutheran General Hospital, Park Ridge, USA

**Keywords:** dlbcl, lymphoma, chop, epoch, non-hodgkin

## Abstract

Diffuse large B-cell lymphoma (DLBCL) represents around one quarter of non-Hodgkin lymphomas in both the United States and globally. The activated B-cell (ABC) subtype of DLBCL is associated with higher relapse rates and a worse prognosis when treated with standard regimens in comparison to other subtypes of DLBCL. Recent studies have demonstrated a potential benefit with combination of dose-adjusted rituximab, etoposide, prednisone, vincristine, cyclophosphamide, and doxorubicin (DA-REPOCH) in comparison to standard combination chemotherapy with cyclophosphamide, doxorubicin, vincristine, and prednisone (CHOP) in ABC DLBCL patients. We aimed to see if there was any benefit on progression-free survival (PFS) and overall survival (OS) in a pooled patient population from a community oncology practice with the use of DA-REPOCH in ABC DLBCL. Our study did not reveal a statistically significant advantage in either PFS or OS with DA-REPOCH; however, a smaller percentage or patients progressed or relapsed when treated with DA-REPOCH. While the toxicity profile was similar, a higher percentage of patients receiving R-CHOP experienced grade 3 or higher toxicities. A prospective trial of R-CHOP versus DA-REPOCH in patients with the ABC subtype of DLBCL is warranted to further determine a potential benefit to DA-REPOCH in this patient population.

## Introduction

Diffuse large B-cell lymphoma (DLBCL) is the most common subtype of non-Hodgkin lymphoma, representing 21% of all non-Hodgkin lymphomas diagnosed in United States from 2008 to 2017 [1]. Combination chemotherapy with cyclophosphamide, doxorubicin, vincristine, and prednisone (CHOP) became the standard of care for DLBCL based on the results of national cooperative group trials conducted in the 1970s and 1980s [2,3]. Treatment of aggressive lymphomas with CHOP resulted in complete response rates of 45%-55% and cured approximately 30%-40% of patients [2,4,5]. CHOP remained the standard of care for these patients throughout the 1990s, despite the development of later generation combination chemotherapy regimens such as methotrexate, bleomycin, doxorubicin, cyclophosphamide, vincristine, dexamethasone (m-BACOD); prednisone, methotrexate, doxorubicin, cyclophosphamide, etoposide plus cytarabine, bleomycin, vincristine, methotrexate (ProMACE-CytaBOM); and methotrexate, doxorubicin, cyclophosphamide, vincristine, prednisone (MACOP) as these regimens did not improve response rates or survival and were more toxic [4]. The addition of rituximab to standard CHOP therapy (R-CHOP) in the early 2000s was a major stride in the treatment of DLBCL as it resulted in improved response rates and survival compared to CHOP alone, without significant toxicity [5,6].

Today, R-CHOP cures 50%-60% of patients with DLBCL, but many patients are either refractory to R-CHOP or will relapse after obtaining a complete remission [7]. It remains critically important to identify high-risk features that predict failure of R-CHOP and necessitate alternative treatment regimens. Gene expression profiling and subsequent immunohistochemistry analysis have stratified diffuse large B-cell lymphoma into germinal center and non-germinal center subtypes [7]. The non-germinal center subtype includes the activated B-cell (ABC) subtype [7].

It is now well-established that patients with the ABC subtype of DLBCL have inferior outcomes when treated with R-CHOP compared to those with the germinal center subtype of the disease. Gutiérrez-García et al. reported that among 157 patients with DLBCL treated with rituximab containing regimens, those with the ABC subtype had inferior PFS and OS compared to the germinal center subtype [8]. This trend has been reported by other investigators [9].

As the ABC subtype of DLBCL responds poorly to standard R-CHOP, alternative treatment regimens are needed. Recently, a phase III randomized trial of patients with DLBCL showed no improvement in response rates or progression-free survival (PFS) with a combination of dose-adjusted rituximab, etoposide, prednisone, vincristine, cyclophosphamide, and doxorubicin (DA-REPOCH) compared to R-CHOP [10]. However, the two-year PFS among patients treated with R-CHOP in this trial was better than expected at 75.5%, indicating that high-risk patients such as those with the ABC subtype may have been underrepresented. The optimal treatment for patients with the ABC subtype of DLBCL remains unknown, and further evaluation of the efficacy of DA-REPOCH in this subtype of lymphoma is warranted.

## Materials and methods

We performed a retrospective analysis to investigate outcomes of patients treated for the ABC subtype of DLBCL at two community oncology practices in the suburbs of Chicago, Illinois. To be eligible for inclusion, patients must have been treated from January 1, 2014 through May 31, 2019 with either DA-REPOCH or R-CHOP. Five years were chosen because few patients were being treated with DA-REPOCH in the practice more than five years ago. All included patients were diagnosed with the ABC subtype of DLBCL according to the World Health Organization classification. Patients were not eligible if they had the germinal center subtype of DLBCL, had a history of prior treatment for aggressive or indolent lymphoma, or were treated with regimens other than R-CHOP or DA-REPOCH. All reported toxicities were graded using the Common Terminology Criteria for Adverse Events, version 5.0.

PFS and OS were calculated in months for each patient using Kaplan-Meier actuarial survival curves [11]. PFS was defined as time from the beginning of treatment until disease progression. OS was defined as time from the beginning of treatment until death from any cause. Differences between treatments were calculated using Fisher’s exact test [12].

## Results

Patient characteristics

A total of 108 patients were treated for DLBCL from January 1, 2014 through May 31, 2019. Of those, we identified 21 patients with the ABC subtype who had been treated with either R-CHOP or DA-REPOCH. We reported their survival as of August 1, 2020. The characteristics of these patients at diagnosis can be seen in Table 1.

**Table 1:** Patient Characteristics at Diagnosis R-CHOP, Rituximab, cyclophosphamide, doxorubicin, vincristine, and prednisone. DA-REPOCH, Dose-adjusted rituximab, etoposide, prednisone, vincristine, cyclophosphamide, and doxorubicin. ECOG, Eastern Cooperative Oncology Group performance status. Note: Performance status was not reported for one patient who received R-CHOP.

Characteristics	R-CHOP (n = 12)	DA-REPOCH (n = 9)
Age	No. (%)	
<65 years	1 (8%)	1 (11%)
65-69 years	2 (17%)	2 (22%)
70-75 years	5 (42%)	3 (33%)
75+ years	4 (33%)	3 (33%)
Female sex	6 (50%)	4 (44%)
ECOG Performance Status		
0	5 (42%)	2 (22%)
1	6 (50%)	7 (78%)
2+	0 (0%)	0 (0%)
Stage		
I	0 (0%)	2 (22%)
II	1 (8%)	0 (0%)
III	3 (25%)	2 (22%)
IV	8 (67%)	5 (56%)
International Prognostic Index		
1	0 (0%)	1 (11%)
2	2 (17%)	1 (11%)
3	7 (58%)	5 (56%)
4	3 (25%)	2 (22%)

The median age of the patients was 73 years. More patients in the R-CHOP group were aged 70 years or older compared to the DA-REPOCH group (75% vs. 66%). Similarly, more patients in the R-CHOP group were stage III or IV at diagnosis (92% vs. 78%). The Eastern Cooperative Oncology Group (ECOG) performance status was reported for 20 of the 21 patients at diagnosis. All patients for whom an ECOG performance status was reported had a score of 0 or 1. The international prognostic index scores were reported for all patients and were similar across the two groups.

Efficacy

Of the 12 patients treated with R-CHOP, 10 achieved a complete remission. Two patients progressed, two subsequently relapsed, and one patient died during therapy. Of the nine patients treated with DA-REPOCH, all achieved a complete remission, and one subsequently died from infectious complications. No patients died during treatment with DA-REPOCH. Ultimately, 33% of patients treated with R-CHOP either progressed or relapsed, whereas none of the patients treated with DA-REPOCH progressed or relapsed. To our knowledge, this trend has not been reported by other investigators. Lymphoma or its treatment was responsible for death in three of 12 patients in the R-CHOP group (25%) compared to one of nine patients in the DA-REPOCH group (11%). All patients who progressed or relapsed were either stage III or IV at diagnosis.

**Figure 1:**
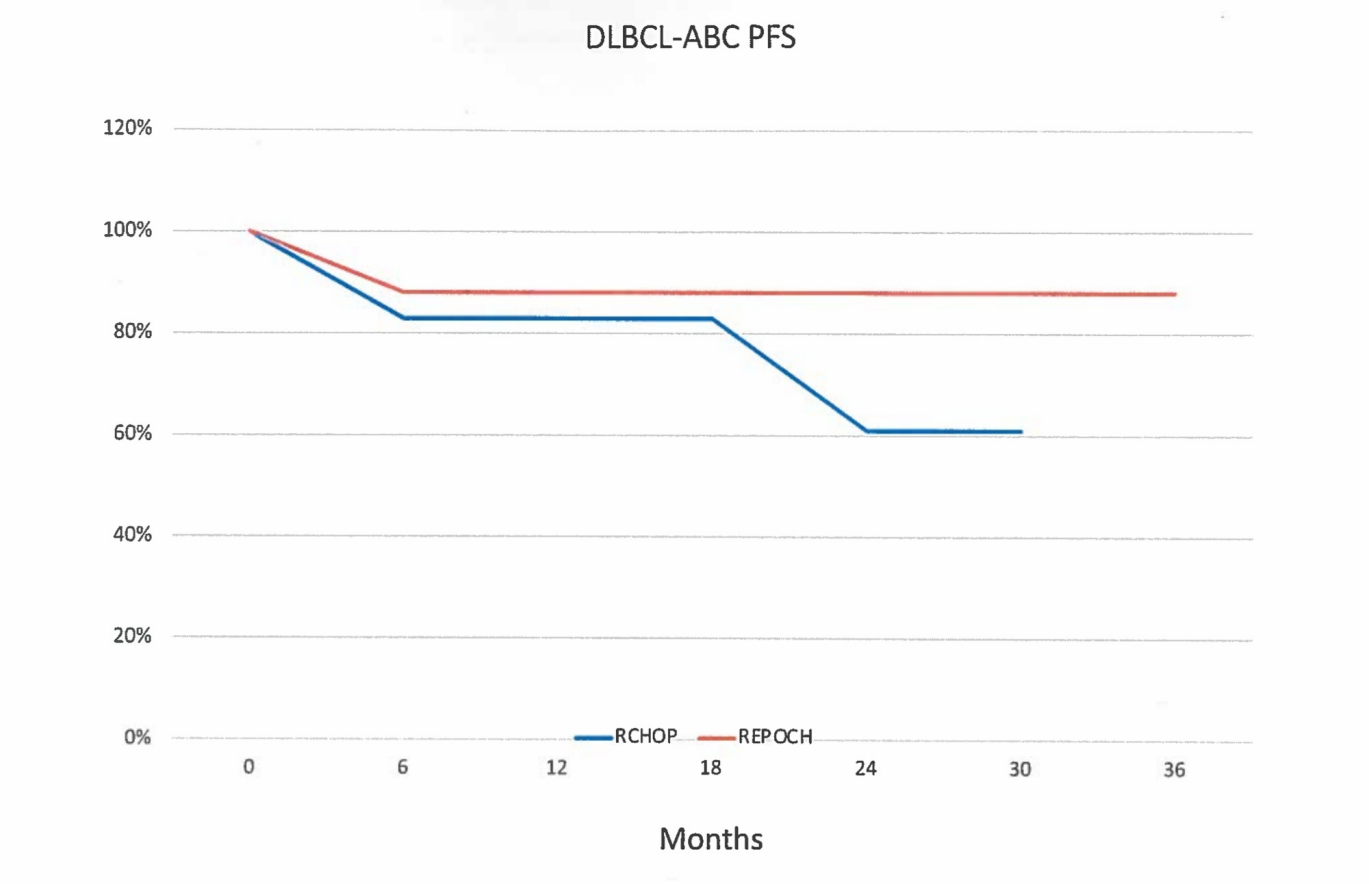
Progression-free survival R-CHOP, Rituximab, cyclophosphamide, doxorubicin, vincristine, and prednisone. DA-REPOCH, Dose-adjusted rituximab, etoposide, prednisone, vincristine, cyclophosphamide, and doxorubicin. DLBCL-ABC, Diffuse large B-cell lymphoma, activated B-cell type. PFS, Progression-free survival.

The three-year actuarial PFS for all 21 patients was 72%. The three-year actuarial PFS for the 12 patients treated with R-CHOP was 61% compared to 88% for the nine patients treated with DA-REPOCH (Figure 1).

This difference was not statistically significant. The three-year actuarial survival for all 21 patients was 88% and was identical in both groups. The three-year PFS is demonstrated on Kaplan-Meier actuarial survival curves in Figure 1.

Toxicity

The toxicities experienced during treatment with R-CHOP and DA-REPOCH are reported in Table 2.

**Table 2:** Toxicities Reported with Treatment R-CHOP, Rituximab, cyclophosphamide, doxorubicin, vincristine, and prednisone. CTCAE, Common Terminology Criteria for Adverse Events. DA-REPOCH, Dose-adjusted rituximab, etoposide, prednisone, vincristine, cyclophosphamide, and doxorubicin. All toxicities reported in our patient population are listed. Toxicities are graded according to CTCAE version 5.

R-CHOP	CTCAE v5 any grade	CTCAE v5 Grade 3 or higher
	No. (% of total population)	
Any	10 (83%)	6 (50%)
Fatigue	6 (50%)	2 (17%)
Peripheral motor neuropathy	3 (25%)	0 (0%)
Heart failure	2 (17%)	2 (17%)
Febrile neutropenia	3 (25%)	3 (25%)
Lung infection	1 (8%)	1 (8%)
Rash	2 (17%)	0 (0%)
Thromboembolic event	1 (8%)	1 (8%)
Sepsis	1 (8%)	1 (8%)
Nausea and vomiting	1 (8%)	1 (8%)
Death	1 (8%)	1 (8%)
DA-REPOCH		
Any	7 (78%)	3 (33%)
Fatigue	4 (44%)	0 (0%)
Peripheral motor neuropathy	1 (11%)	0 (0%)
Generalized muscle weakness	1 (11%)	1 (11%)
Mucositis	1 (11%)	1 (11%)
Nausea and vomiting	1 (11%)	1 (11%)
Death	0 (0%)	0 (0%)

The majority of patients reported some toxicity: 83% of patients treated with R-CHOP and 79% of patients treated with DA-REPOCH. Most toxicities were grade 2 or lower; however, six of 12 patients (50%) treated with R-CHOP experienced grade 3 or higher toxicities, and three of nine patients (33%) treated with DA-REPOCH experienced grade 3 or higher toxicities. The most common toxicities of any grade in both groups were fatigue and peripheral motor neuropathy. The most common grade 3 or higher toxicities in the R-CHOP group were febrile neutropenia, fatigue, and heart failure. The grade 3 toxicities reported in the DA-REPOCH group were generalized muscle weakness, mucositis, and nausea with vomiting. One death was attributed to therapy in the R-CHOP group, whereas no deaths were attributed to therapy in the DA-REPOCH group.

## Discussion

In our retrospective analysis of 21 patients with the ABC subtype of DLBCL, 33% of patients treated with R-CHOP either progressed or relapsed, whereas none of the patients treated with DA-REPOCH progressed or relapsed (p = 0.1038). Additionally, lymphoma or its treatment was responsible for death of 25% of patients treated with R-CHOP compared to only 11% of those treated with DA-REPOCH. Despite these findings, our study did not reveal a statistically significant advantage in either PFS or OS with DA-REPOCH. Surprisingly, the three-year actuarial PFS for all 21 patients was 72%, and the three-year OS was 88%, far better than what has been previously reported for this patient population.

The cancer and leukemia group B (CALGB) 50303 trial published in 2019 investigated frontline DA-REPOCH for the treatment of DLBCL and also found no improvement in either PFS or OS compared to R-CHOP; however, this trial did not specify outcomes of patients with the ABC subtype (10). In contrast to their findings, grade 3 and 4 adverse events were more common in our patients treated with R-CHOP than with DA-REPOCH. One potential explanation for this discrepancy is that patients in the R-CHOP group were more likely to be aged 70 years or older compared to the DA-REPOCH group, though patients were similar in respect to performance status at diagnosis.

There are several limitations to our study. Our sample size is limited as we are reporting treatment outcomes of a relatively rare diagnosis from a single institution. As a result, the baseline patient characteristics were not equally balanced between the two treatment groups as patients treated with R-CHOP were more likely to be over 70 years old and to have a more advanced stage of disease. In addition, our patient population is much older than is typical of most studies of DLBCL.

## Conclusions

In conclusion, our retrospective analysis of 21 patients with the ABC subtype of DLBCL showed that 33% of patients treated with R-CHOP either progressed or relapsed, whereas none of the patients treated with DA-REPOCH progressed or relapsed. Despite these findings, our study did not reveal a statistically significant advantage in either PFS or OS with DA-REPOCH. Surprisingly, the three-year actuarial PFS for all 21 patients was 72%, and the three-year OS was 88%, far better than what has been previously reported for this patient population. Our outcomes are better than those previously reported for this subtype of DLBCL. This difference is difficult to reconcile but may reflect improvement in supportive care. Given our findings, we believe that a prospective trial of R-CHOP versus DA-REPOCH in patients with the ABC subtype of DLBCL is warranted.
